# Effect of Lung Protective Ventilation Combined With Flurbiprofen Axetil on Immune Function During Thoracoscopic Radical Resection of Lung Cancer

**DOI:** 10.3389/fsurg.2022.840420

**Published:** 2022-02-17

**Authors:** Jia Yuan, Shenghua Cen, Jingjing Li, Kun Wang, Qixu Chen, Hongbin Li, Yan Zhang

**Affiliations:** Department of Anesthesiology, Zhoushan Hospital, Zhoushan, China

**Keywords:** lung cancer, lung protective ventilation, flurbiprofen axetil, thoracoscopic radical resection, immunologic function

## Abstract

The decreased immune function of patients with lung cancer has always been the focus of clinical attention. However, the stress response caused by surgery, anesthesia and pain will further reduce the body's immune function and affect the prognosis of patients to a certain extent. It was found that both protective ventilation and flurbiprofen ester pretreatment could reduce the immunosuppression caused by stress response. In this study, 120 lung cancer patients treated with video-assisted thoracoscopic radical resection were divided into group A, group B, group C and group D, which were treated with conventional mechanical ventilation, lung protective ventilation, conventional mechanical ventilation + flurbiprofen axetil and lung protective ventilation + flurbiprofen axetil, respectively. The results showed that the levels of CD3+, CD4+, CD4/CD8+, and NK in groups A, B, and C were lower than T0 on T1, T2, and T3, while those indicators in group D were lower than T0 on T1 and T2 (*P* < 0.05). The above indicators in group D were higher than those in the other three groups on T1, T2, and T3 (*P* < 0.05). The above indicators were statistically significant compared with those in group A and group C, group B and group D, and group A and group B at T1, T2, and T3 (*P* < 0.05). The comparisons of CD3+, CD4+, CD4/CD8+, and NK among the four groups within different time groups, and the repeated - measures analysis of variance (repeated - measures ANOVA) showed that there were interactions among time, group, and between groups × within groups (*P* < 0.05). It was confirmed that lung protective ventilation combined with flurbiprofen axetil could alleviate the immunosuppression of patients undergoing thoracoscopic radical lung cancer, providing a new idea for clinical treatment.

## Introduction

Lung cancer can be divided into two types of non-small cell lung cancer (NSCLC) and small cell lung cancer (SCLC), and 80% of patients with NSCLC. The 5-year survival rate of lung cancer patients is low, and in recent years, the onset age of lung cancer patients also tends to be younger (1). Thoracoscopic radical resection of lung cancer is currently an important means of treatment for lung cancer. But it needs to be performed under mechanical ventilation, which has a certain inhibitory effect on patients' autoimmunity, while narcotic drugs (especially opioids) also have an impact on immunity (2, 3). However, immunity is closely related to the patient's ability to resist external pathogen infection, and the balance regulation of immune system also plays an important role in maintaining body homeostasis. Once the immune balance is out of balance, the body is very prone to infection, and autoimmune diseases. Therefore, alleviating the immunosuppressive effects of mechanical ventilation and anesthesia is of great significance for improving the prognosis of patients undergoing video-assisted thoracoscopic radical resection of lung cancer (4).

In recent years, studies have pointed out that lung protective ventilation and flurbiprofen ester can weaken the inhibition of the above factors on the immune system of patients. On this basis, we hereby studies the effect of combination of the two on immune function in patients undergoing radical lung cancer surgery, as reported below.

## Materials and Methods

### General Data

One hundred twenty patients with lung cancer who received thoracoscopic radical resection of lung cancer in our hospital from April 2017 to June 2020 were included as research objects and divided into 4 groups. The ages of group A, B, C, and D were (56.15 ± 3.65) years old, (57.24 ± 3.95) years old, (55.78 ± 7.21) years old, and (57.16 ± 3.65) years old. Male and female composition: 19/11, 17/13, 18/12, and 19/11; ASAi/ii constituted 6/24, 2/28, 7/23 and 4/26; BMI was (23.15 ± 2.39) kg/m^2^, (23.36 ± 3.01) kg/m^2^, (22.96 ± 2.57) kg/m^2^ and (22.37 ± 5.14) kg/m^2^. The mean course of disease was (6.17 ± 1.52) months, (6.25 ± 1.37) months and (6.33 ± 1.29) months. There was no difference in the general data of the four groups(*P* > 0.05).

### Inclusion Criteria

i) 18–69 years old; ii) The BMI was 18–28 kg/m^2^; iii) Meeting the Class I–II criteria of the American Association of Anesthesiologists (5); iv) Sign informed consent form.

### Exclusion Criteria

i) Combined with fever, cough and gastrointestinal ulcers; ii) Patients who took NSAIDs for a long time before entering the group; iii) Patients with combined history of consciousness disorder and mental disease; iv) Patients with pulmonary tuberculosis and bronchial asthma; v) Patients requiring blood transfusion during the operation; vi) Combined with kidney, liver, heart and other major organ dysfunction; vii) Taking glucocorticoids and immunosuppressive agents before operation; viii) Patients with coagulation abnormalities and severe endocrine diseases; ix) Allergic to drugs used in this study; x) The pathological type of lung cancer is not suitable for thoracoscopic lung cancer radical surgery.

### Methods of Anesthesia and Mechanical Ventilation

Routine anesthesia: 0.5 mg penehycliane hydrochloride was intravenously injected 30 min before surgery, oxygen was inhaled via nasal catheter, peripheral venous access of the upper limb was opened, SpO_2_, HR and other indicators were detected. The non-operative radial artery puncture was performed under local anesthesia, and the invasive blood pressure was monitored. The lateral internal jugular vein puncture was completed under local anesthesia ultrasound guidance, and the CVP was maintained within the range of 5–10 cmH_2_O. Midazolam, etomidate, sufentanil and rocuronium were given intravenously at doses of 0.05 mg/kg, 0.2 mg/kg, 0.4 ug/kg and 0.8 mg/kg. Indwelling of the left double-lumen bronchocatheter was performed under laryngoscopy. Under the assistance of a laryngoscope, the left double-cavity bronchial catheter was placed. Localization was performed by fiberoptic bronchoscopy. Mechanical ventilation was performed with A5 anesthesia, and volumetric controlled ventilation mode was used. After the patient's position was changed, the indwelling position of the double lumen tube was observed again.

Group A underwent conventional ventilation: one-lung ventilation was performed at Vr8ml/kg and RR13–16 times/min; The bilateral lung ventilation rate was Vr10ml/kg, and RR10–12 times /min. Group B was treated with protective ventilation: One-lung ventilation was given at Vr6ml/kg and RR14–16 times/min. Two-lung ventilation was given at Vr8ml/kg and RR12–14 times /min. One-lung ventilation was performed with PEEP5 cmH_2_O, oxygen flow rate of 1–2 L/min, I: E ratio of 1:2, and FIO 2100%. PETCO_2_ was maintained at 35–45 mmHg. Anesthesia was maintained by target controlled infusion of remifentanil and propofol, with target plasma concentrations of 2–4 ng/mL and 2–4 mg/mL, respectively. During infusion, the dosage and infusion speed were adjusted according to the arterial pressure to maintain the arterial pressure fluctuation to be ≤ 20% of the preoperative level. Before skin incision, 0.2 ug/kg sufentanil was given intravenously, while 0.05 mg/kg atracurium besylate was given intermittently during the process, to maintain the Narcotrend index within the range of 37–64. Also, 6 mL kg/h compound sodium lactate was given intravenously during the operation. All patients stopped drug administration at the completion of the operation. After the patients were conscious and the muscle strength recovered, the double-lumen endobronchial tube was removed. Meanwhile, the same scheme of analgesic pump was used for analgesia within 24 h after the operation. In addition, in the groups C and B, flurbiprofen axetil 2 mg/Kg was given intravenously 5 min before anesthesia induction.

### Observation Indicators

T0-t4 was used to represent preoperative, post-operative, postoperative 24 h, 72 h, and 7 d. At the above time, the expression of CD3+, CD4+, CD8+ and NK cells was detected by FC500 flow cytometry, and CD4+/CD8+ was calculated. 2 ml venous blood was taken in the fasting state in the morning, and heparin anticoagulant blood was taken (1: 9) 100P1 was added with monoclonal antibody, kept away from light for 12 min at room temperature, centrifuged, washed twice by PBS, and then added with 0.5 mL 1% paraformaldehyde. Machine detection was performed. Homotype negative control was performed for each sample at the same time. The number of cells per sample was 10,000, and the percentage of positive cells was calculated.

### Statistical Methods

All data were processed with SPSS 22.0 statistical software, and GraghPad prism 8 was used to make statistical graphs. Measurement data are expressed as mean ± standard deviation (X̄± s), the comparisons of four groups at different times were performed with repeated measures analysis of variance and F test. The count data between groups were expressed in percentage (%) and tested by “x^2^”. The difference is statistically significant when *P* < 0.05.

## Results

### Comparison of CD3+ Expression

CD3+ in Groups A, B, and C was lower than T0 on T1, T2, and T3, while it was lower than T0 on T1 and T2 in Group D; T1, T2, and T3 in group D were higher than those of the other three groups from T1 to T3. The CD3+ expressions on T1, T2, and T3 in group A and group C, group B and group D, and group A and group B were all statistically significant (*P* < 0.05). Repeated measures analysis of variance: F_time_ = 121.201 (*P* < 0.001); F_Group_ = 76.951 (*P* < 0.001); F_Time × *grouping*_ = 65.150 (*P* < 0.001, Table 1).

**Table 1:** Comparison of CD3+ expression among four groups (X̄± s).

**Group**		**T0**	**T1**	**T2**	**T3**	**T4**
Group A	30	65.52 ± 6.51	43.62 ± 5.36^a^	49.65 ± 7.75^a^	54.65 ± 6.97^a^	63.98 ± 8.98
Group B	30	65.15 ± 6.98	50.52 ± 8.65^a^	56.21 ± 6.99^a^	60.36 ± 5.61^a^	65.15 ± 5.87
Group C	30	64.75 ± 8.62	48.65 ± 7.16^a^	54.12 ± 5.98^a^	58.62 ± 5.67^a^	64.54 ± 8.64
Group D	30	66.65 ± 7.18	57.65 ± 5.49^a^	60.54 ± 9.65^a^	65.98 ± 7.69	65.78 ± 8.34
F/P	-	2.575/0.462	21.90/0.000	10.33/0.000	15.50/0.000	0.28/0.841
Q/P	A and B	0.275/>0.05	5.557/ <0.01	4.656/ <0.01	5.7101/ <0.01	0.796/>0.05
Q/P	A and C	0.573/>0.05	4.052/ <0.01	3.171/ <0.01	3.970/ <0.01	0.381/>0.05
Q/P	A and D	0.840/>0.05	11.301/ <0.01	7.731/ <0.01	11.330/ <0.05	1.225/>0.05
Q/P	B and C	0.298/>0.05	1.506/>0.05	1.485/>0.05	1.740/>0.05	0.415/>0.05
Q/P	B and D	1.116/>0.05	5.743/ <0.01	3.076/ <0.01	5.620/ <0.01	0.429/>0.05
Q/P	C and D	1.413/>0.05	7.249/ <0.01	4.561/ <0.01	7.360/ <0.01	0.844/>0.05

*Comparison with T0*,

a *P < 0.05*.

### Comparison of CD4+ Expression

CD4+ in group A, B and C was lower than T0 at T1, T2, and T3, and lower than T0 at T1 and T2 in group D. The CD4+ of group D was higher than that of the other three groups at T1, T2, and T3. The expression of CD4+ in group A and GROUP C, group B and group D, and group A and group B was statistically significant at T1, T2, and T3 (*P* < 0.05). The comparison of CD4+ in group A and group B at T1, T2, and T3 was statistically significant (*P* < 0.05). Anova of repeated measures: F_time_ =89.113 (*P* < 0.001); F_Group_ = 89.658 (*P* < 0.001); F_Time × *grouping*_=41.625 (*P* < 0.001, Table 2).

**Table 2:** Comparison of CD4+ expression among the four groups (X̄± s).

**Group**		**T0**	**T1**	**T2**	**T3**	**T4**
Group A	30	39.12 ± 5.98	26.54 ± 4.98^a^	28.13 ± 3.65^a^	31.26 ± 4.54^a^	37.54 ± 9.54
Group B	30	39.49 ± 6.21	31.32 ± 5.15^a^	33.35 ± 5.65^a^	34.65 ± 5.45^a^	38.32 ± 6.67
Group C	30	39.06 ± 7.11	29.65 ± 3.54^a^	31.23 ± 7.45^a^	34.59 ± 5.18^a^	38.27 ± 7.14
Group D	30	38.95 ± 6.98	34.54 ± 5.24^a^	36.57 ± 5.65^a^	38.67 ± 6.87	38.14 ± 8.03
F/P	-	0.04/0.9901	14.63/0.000	11.42/0.000	8.87/0.000	0.06/0.980
Q/P	A and B	0.308/>0.05	5.480/ <0.01	4.965/ <0.01	3.330/ <0.05	0.539/>0.05
Q/P	A and C	0.05/>0.05	3.565/ <0.01	2.948/ <0.05	3.271/ <0.05	0.505/>0.05
Q/P	A and D	0.141/>0.05	9.171/ <0.01	8.027/ <0.01	7.279/ <0.01	0.415/>0.05
Q/P	B and C	0.358/>0.05	1.914/>0.05	2.016/>0.05	0.059/>0.05	0.035/>0.05
Q/P	B and D	0.449/>0.05	3.691/ <0.01	3.062/ <0.05	3.949/ <0.01	0.125/>0.05
Q/P	C and D	0.092/>0.05	5.606/ <0.01	5.079/ <0.01	4.008/ <0.05	0.090/>0.05

*Comparison with T0*,

a *P < 0.05*.

### Comparison of CD4+/CD8+ Expression

CD4+/CD8+ in groups A, B and C were lower than T0 on T1, T2, and T3, while CD4+/CD8+ in group D was lower than T0 on T1 and T2. Group D had higher CD4+/CD8+ values on T1, T2, and T3 than the other three groups. The comparisons of T1, T2, and T3 between group A and group C, group B and group D, and group A and group B were statistically significant (*P* < 0.05). Repeated measures analysis of variance: F_time_ = 69.067 (*P* < 0.001); F_Group_ = 49.167 (*P* < 0.001); F_Time × *grouping*_ = 29.117 (*P* < 0.001, Table 3).

**Table 3:** Comparison of CD4+/CD8+ expression among four groups (X̄± s).

**Group**		**T0**	**T1**	**T2**	**T3**	**T4**
Group A	30	2.51 ± 1.32	1.30 ± 0.39^a^	1.40 ± 0.59^a^	1.62 ± 0.41^a^	2.47 ± 0.58
Group B	30	2.52 ± 1.49	1.65 ± 0.57^a^	1.75 ± 0.32^a^	2.16 ± 0.39^a^	2.56 ± 0.52
Group C	30	2.59 ± 0.95	1.52 ± 0.32^a^	1.72 ± 0.31^a^	2.03 ± 0.40^a^	2.50 ± 0.59
Group D	30	2.60 ± 0.57	1.99 ± 0.36^a^	2.36 ± 0.29^a^	2.46 ± 0.39	2.58 ± 0.38
F/P	-	0.05/0.985	14.11/0.000	30.59/0.000	23.04/0.000	0.29/0.835
Q/P	A and B	0.048/>0.05	4.553/ <0.01	4.828/ <0.01	7.439/ <0.05	0.940/>0.05
Q/P	A and C	0.385/>0.05	2.862/ <0.01	4.414/ <0.05	5.648/ <0.05	0.313/>0.05
Q/P	A and D	0.433/>0.05	8.977/ <0.01	13.242/ <0.01	11.572/ <0.01	1.149/>0.05
Q/P	B and C	0.337/>0.05	1.691/>0.05	0.414/>0.05	1.791/>0.05	0.627/>0.05
Q/P	B and D	0.385/>0.05	4.423/ <0.01	8.414/ <0.05	4.133/ <0.01	0.209/>0.05
Q/P	C and D	0.048/>0.05	6.115/ <0.01	8.828/ <0.01	5.924/ <0.05	0.836/>0.05

*Comparison with T0*,

a *P < 0.05*.

### Comparison of NK Expression

NK in group A, B and C was lower than T0 at T1, T2, and T3, and NK in group D was lower than T0 at T1 and T2. NK in group D was higher at T1, T2, and T3 than in the other three groups. There were statistically significant differences between group A and GROUP C, group B and group D, group A and group B at T1, T2, and T3 (*P* < 0.05). Anova of repeated measures: F_time_ = 59.621 (*P* < 0.001); F_Group_ = 39.651 (*P* < 0.001); F_Time × *grouping*_ = 23.780 (*P* < 0.001, Table 4).

**Table 4:** Comparison of NK expression among four groups (X̄± s).

**Group**		**T0**	**T1**	**T2**	**T3**	**T4**
Group A	30	22.10 ± 0.85	11.03 ± 1.65^a^	13.86 ± 1.32^a^	16.02 ± 1.36^a^	22.03 ± 1.36
Group B	30	22.03 ± 0.74	14.52 ± 1.36^a^	16.26 ± 1.47^a^	20.03 ± 2.03^a^	22.42 ± 2.45
Group C	30	21.98 ± 0.69	14.03 ± 1.59^a^	17.36 ± 2.08^a^	19.65 ± 1.99^a^	21.89 ± 1.98
Group D	30	22.19 ± 0.97	17.62 ± 1.49^a^	20.36 ± 3.65^a^	23.92 ± 2.46	22.80 ± 0.65
F/P	–	0.37/0.774	93.72/0.000	40.50/0.000	78.32/0.000	1.65/0.181
Q/P	A and B	0.468/>0.05	12.523/ <0.01	5.663/ <0.01	10.988/ <0.01	1.223/>0.05
Q/P	A and C	0.802/>0.05	10.765/ <0.01	8.259/ <0.01	9.947/ <0.01	0.439/>0.05
Q/P	A and D	0.601/>0.05	23.646/ <0.01	15.338/ <0.01	21.647/ <0.01	2.415/>0.05
Q/P	B and C	0.334/>0.05	1.758/>0.05	2.596/>0.05	1.041/>0.05	1.663/>0.05
Q/P	B and D	1.069/>0.05	11.123/ <0.01	9.675/ <0.05	10.659/ <0.01	1.192/>0.05
Q/P	C and D	1.403/>0.05	12.882/ <0.01	7.079/ <0.01	11.700/ <0.05	2.855/>0.05

*Comparison with T0*,

a *P < 0.05*.

## Discussion

### Deficiencies of Conventional Mechanical Ventilation

Mechanical ventilation is an important supportive treatment in ICU. It can not only effectively maintain the airway patency of patients, improve oxygenation and ventilation, but also prevent carbon dioxide accumulation and hypoxia in the body, thereby enabling the body to avoid respiratory failure caused by basic lesions. However, many studies have confirmed that within 2–4 h of conventional mechanical ventilation, the susceptibility of patients to bacteremia is significantly higher than that of patients without mechanical ventilation. Excessive mechanical ventilation results in the accumulation of cytokines, white blood cells, and neutrophil-dependent tissue damage, resulting in cell activation and release of mediators, leading to alveolar inflammation (6). In addition, the observation of NK cell expression during conventional mechanical ventilation (10 ml/kg tidal volume) in infants without pulmonary diseases undergoing cardiac catheterization also showed that the activity of NK cells in peripheral blood began to decrease 2 h after the operation. The subjects of this study were lung cancer patients, and the results showed that the levels of CD3+, CD4+, CD4/CD8+, and NK in the four groups at T1 were lower than those at T0, which confirm that routine mechanical ventilation can adversely affect the patient's immune system.

### Application of Lung Protective Ventilation

Lung protective ventilation strategies include appropriate PEEP and low tidal volumes (7). In animal experiments, the expression of NK cells in peripheral blood of mice with different mechanical ventilation schemes was compared and analyzed after 4 h of ventilation. It was found that high tidal volume ventilation could cause significant immunosuppression, and the decline degree of NK cells in mice with high tidal volume and without PEEP was more significant than that with high tidal volume and PEEP. And the combination of low tidal volume and PEEP could alleviate the immunosuppression caused by mechanical ventilation (8). In this study, the expression levels of the above indicators in group A were lower than those in group B from T1 to T3 (*P* < 0.05). This is due to compared with conventional mechanical ventilation, lung protective ventilation can alleviate alveolar-capillary barrier damage and inhibit inflammatory response. It was reported that 90% of patients with general anesthesia can appear atelectasis. During general anesthesia, low pulmonary volume ventilation can lead to repeated collapse and reopening of the alveolar space, which further affects small airway epithelial cells, leading to the occurrence of atelectasis (9, 10). Driven by experimental and clinical studies, mechanical ventilation can reduce tidal volume and limit lung dilation to a certain extent. Previous studies have pointed out that when tidal volume is 15 ml/kg, end-expiratory lung volume can be improved and intraoperative atelectasis can be relieved (11). In addition, if there are no contraindications, the use of positive end-expiratory pressure and lung recruitment can also help prevent end-expiratory lung volume loss and small airway closure during anesthesia (12). Although 10 ml/kg tidal volume was mostly used in clinical practice in the past, anesthesiologists would reduce tidal volume during single ventilation. Moreover, many studies have pointed out that a tidal volume of 4–5 mL/kg can better protect lung tissue while fully satisfying the gas exchange (13). The tidal volume selected for lung protective ventilation in this study belongs to the safe range (14).

### Flurbiprofen Ester Alleviates Immunosuppression

Flurbiprofen ester is a non-steroidal analgesic drug, which can inhibit coX-2 release, prostaglandin synthesis, inflammatory factor release and other mechanisms through selective aggregation in surgical incision and inflammatory tissue, and exert targeted analgesic effect. It can reduce the dose of opioids, and is currently mainly used for cancer pain treatment, postoperative analgesia and preemptive analgesia et al. (15, 16). In addition, compared with tramadol or morphine, flurbiprofen had the weakest immunosuppressive effect during postoperative analgesia (17). Previous studies have indicated that postoperative analgesia with flurbiprofen axetil can alleviate postoperative immunosuppression, and protect the immune function of cancer patients (18, 19). Zhang et al. (20) pointed out that the decrease of CD4+, CD3+, CD4+/CD8+ and NK cell activity in sufentanil combined with flurbiprofen ester was lower than that in sufentanil alone. Anova of this study showed that group and time had impact on each indicator (*P* < 0.001), which suggesting that flurbiprofen ester could relieve immunosuppression caused by anesthesia or surgery.

In summary, lung protective ventilation combined with flurbiprofen axetil in video-assisted thoracoscopic lung cancer radical surgery can alleviate immunosuppression and facilitate postoperative recovery, which is worthy of promotion.

## Data Availability Statement

The original contributions presented in the study are included in the article/supplementary material, further inquiries can be directed to the corresponding author/s.

## Ethics Statement

The studies involving human participants were reviewed and approved by the Ethics Committee of Zhoushan Hospital of Wenzhou Medical University. The patients/participants provided their written informed consent to participate in this study.

## Author Contributions

JY and SC are mainly responsible for the writing of the paper. JL is responsible for the design of the research. KW and QC are responsible for the detection and evaluation of the results. HL is responsible for data recording and statistics. YZ is the instructor of the entire research. All authors contributed to the article and approved the submitted version.

## Funding

This work was funded the Department of Science and Technology Program in Zhejiang Province (2018C37134) and the Health Commission of Zhejiang Province (2018KY855).

## Conflict of Interest

The authors declare that the research was conducted in the absence of any commercial or financial relationships that could be construed as a potential conflict of interest.

## Publisher's Note

All claims expressed in this article are solely those of the authors and do not necessarily represent those of their affiliated organizations, or those of the publisher, the editors and the reviewers. Any product that may be evaluated in this article, or claim that may be made by its manufacturer, is not guaranteed or endorsed by the publisher.
